# Unpacking the impact of chronic pain as measured by the impact stratification score

**DOI:** 10.1186/s12891-022-05834-4

**Published:** 2022-09-23

**Authors:** Anthony Rodriguez, Maria Orlando Edelen, Patricia M. Herman, Ron D. Hays

**Affiliations:** 1 RAND Corporation, Behavioral and Policy Sciences, 20 Park Plaza #920, Boston, MA USA; 2 Patient Reported Outcomes, Value and Experience (PROVE) Center, Department of Surgery, Brigham and Women’s Hospital, Boston, MA USA; 3 RAND Corporation, Behavioral and Policy Sciences, 1776 Main Street, Santa Monica, CA USA; 4 Division of General Internal Medicine & Health Services Research, UCLA Department of Medicine, Los Angeles, CA USA

**Keywords:** Chronic low back pain, Impact stratification, PROMIS®, Reliability, Bifactor, Patient-reported outcomes

## Abstract

**Background:**

In 2014, the National Institute of Health Pain Consortium’s research task force on research standards for chronic low back pain (CLBP) proposed a measure that could be used to stratify patients by the impact CLBP has on their lives, namely the Impact Stratification Score (ISS). This study examines the dimensionality of the ISS and support for its single total score, and evaluates its overall psychometric properties.

**Methods:**

The sample included 1677 chiropractic patients being treated for CLBP and chronic neck pain, had an average age of 49, 71% female, and 90% White. Study participants completed the PROMIS-29 v2.1 profile survey that contains the 9 ISS items. The ISS was evaluated using item-total correlations, Cronbach’s alpha, factor analysis (i.e., correlated factors and bifactor models), and item response theory (IRT). Reliability indices and item properties were evaluated from bifactor and IRT models, respectively.

**Results:**

Item-total correlations were high (0.64–0.84) with a Cronbach’s alpha of 0.93. Eigenvalues suggested the possibility of two factors corresponding to physical function and pain interference/intensity. Bifactor model results indicated that data were essentially unidimensional, primarily reflecting one general construct (i.e., impact) and that after accounting for ‘impact’ very little reliable variance remained in the two group factors. General impact scores were reliable (omegaH = .73). IRT models showed that items were strong indicators of impact and provided information across a wide range of the impact continuum and offer the possibility of a shorter 8-item ISS. Finally, it appears that different aspects of pain interference occur prior to losses in physical function.

**Conclusions:**

This study presents evidence that the ISS is sufficiently unidimensional, covers a range of chronic pain impact and is a reliable measure. Insights are obtained into the sequence of chronic pain impacts on patients’ lives.

**Supplementary Information:**

The online version contains supplementary material available at 10.1186/s12891-022-05834-4.

## Background

The 2011 Institute of Medicine (IOM) report Relieving Pain in America established the prevalence and multidimensionality of chronic pain impact [1]. The report also called for a cultural transformation regarding the diagnosis and treatment of pain. For years pain was considered a symptom measurable using a single pain intensity item [2]. However, this report was key in introducing the broader construct of the “impact” of chronic pain. “No simple clinical test can assess a person’s subjective experience of pain. Seriousness depends on self-report and to some extent can be inferred from pain’s impact on a person’s activities of daily living, ability to work, and quality of life” [1], p86.

As a result of the IOM report, the National Pain Strategy (NPS) was published in 2015 that introduced a focus on patients with high-impact chronic pain [3]. The report defined high-impact chronic pain as being: “associated with substantial restriction of participation in work, social, and self-care activities for six months or more” [3], p11. Further it went on to say “It is important to differentiate people with high-impact chronic pain from those who maintain normal activities although experiencing chronic pain” [3], p17. The NPS population research working group stated that it was essential to identify those with high-impact chronic pain because they: “account for a large share of the societal costs of chronic pain, and they bear the greatest personal costs” [4], p1070.

The personal and societal costs of high-impact chronic pain have been shown in many studies. Those with high-impact versus milder levels of chronic pain have significantly greater healthcare utilization and higher healthcare costs; [4–8] more unemployment and absenteeism; [6, 7] more opioid use; [6, 8] worse depression; [6] and lower health utility (i.e., societal preference for the health state [9]) [7].

In 2014, the National Institute of Health Pain Consortium’s research task force (RTF) on research standards for chronic low back pain (CLBP) proposed a measure that could be used to stratify patients by the impact CLBP has on their lives [10]. This measure, the impact stratification score (ISS), was constructed using a subset of items contained in the Patient-Reported Outcomes Measurement Information System (PROMIS®) 29-item profile measure (PROMIS-29). The PROMIS-29 assesses seven health domains with 4 items each (Physical Function, Pain Interference, Fatigue, Sleep Disturbance, Depression, Anxiety, and Social Role Functioning), and also includes a single item to assess pain intensity. Specifically, the ISS is constructed as the sum of the items from the PROMIS-29 that assess physical function (4 items, each scored 1–5), pain interference (4 items, each scored 1–5), and pain intensity (1 item scored 0–10), resulting in a score with a possible range from 8 (least impact) to 50 (greatest impact).

Although the RTF provided some tentative cutoff points for using the ISS for stratification, evaluation of the ISS to date has focused on it as a continuous measure. The RTF used a sample of 218 patients with LBP who received epidural steroid injections to examine the validity of the ISS [10]. The ISS was highly correlated with the Roland-Morris Disability Questionnaire, RMDQ (0.66) and the Oswestry Disability Index, ODI (0.81) at baseline, and more responsive to change than the RMDQ. Assuming that the RMDQ and the ODI measure at least some aspects of impact, these results were promising.

Despite the RTF recommendation for further assessment of the reliability, validity and clinical utility of the ISS, we found only two other published studies that attempted this. In a study of 198 patients with chronic musculoskeletal pain and pain intensity of 5 or greater on a 0–10 scale [11] the ISS had an internal consistency reliability of 0.91, an intraclass correlation coefficient of 0.73 among patients who said their pain was “about the same” at 3-months follow up (test–retest reliability), and the ISS was monotonically higher across patients’ statements as to how much worse their pain was at 3 months. Also, Cronbach’s alpha and kappa values were generally similar for those with CLBP and those with other musculoskeletal pain conditions. Another study of 223 spine center patients with CLBP and/or chronic leg pain estimated the minimal clinically important change for the ISS as 7.5 points [12].

This paper adds to the work done to date to evaluate the reliability and clinical usefulness of the ISS as a measure of the impact of CLBP. Because the ISS is the simple sum of nine items, 4 from each of two well-studied PROMIS scales plus a single pain intensity item, we examine its overall dimensionality (i.e., can it be considered a continuous, unidimensional measure of impact?), the appropriateness of combining the raw item scores into a total score, and further characterize the psychometric properties of the ISS items. Lastly, we evaluate the construct validity of the ISS by examining its association with a legacy pain measure.

## Method

### Data source

The Center for Excellence in Research for Complementary and Integrative Health (CERC) [13] data were collected longitudinally on a US sample of chiropractic patients being treated for CLBP and chronic neck pain (CNP) and included three subgroups: CLBP only (*n* = 518), CNP only (*n* = 347), and CLBP + CNP (*n* = 1159). The study was approved by the Human Subject Protection Committee at the RAND Corporation. Baseline data were used for all analyses and we excluded those indicating the presence of CNP only, resulting in a sample of 1677 with CLBP. The mean age of the 1677 respondents was 49 (SD = 15) ranging from 21 to 95 years of age and 71% were female. The sample was predominantly White (90%) with relatively low rates for Black (2%), Asian (3%), Pacific Islander (0.5%), American Indian (2%), Native Alaskan (4%) and Other (1%). Hispanic ethnicity was indicated by 4% of the sample.

### Measures

The PROMIS-29 v. 2.1 instrument includes the 9-item ISS: four items assessing physical function (PF; ability to perform physical activities including chores around the house, climbing stairs, walking, and instrumental activities of daily living, such as running errands), with item responses coded from 1 (without any difficulty) to 5 (unable to do) with higher scores indicating poorer functioning; [14] four items assessing pain interference (PI; the extent to which pain hinders engagement with day-to-day activities, social activities, chores, and work around the home), with item responses ranging from 1 (not at all) to 5 (very much) and higher scores indicating more pain interference; [15] and, a single pain intensity item reflecting the intensity of pain a person experienced, on average over the past 7 days on a scale from 0 (no pain) to 10 (worst pain imaginable) with higher scores indicating greater pain intensity.

The Oswestry Disability Index (ODI) is a 10-item measure assessing pain intensity, personal care, lifting, walking, sitting, standing, sleeping, sex life (if applicable), social life, and traveling. Response options range from 0 to 5 with higher scores indicating greater disability. The scale is scored by summing scores across all items, dividing the total score by the maximum possible and then multiplying by 100. The ODI score can also be classified into five severity groups [16].

### Analyses

The goals of this study were to: 1) examine the dimensional structure of the ISS in a CLBP sample; 2) determine the appropriateness of combining the nine items to form a single composite total score; and, 3) if the ISS is found to be sufficiently unidimensional, further evaluate the properties of ISS items. This is particularly of interest because of the presence of content clusters (PF and PI) in the nine ISS items that may pose a threat to unidimensionality. We first examine correlations among all items, item-test correlations (correcting for item overlap with the total score), and Cronbach’s alpha. Next, we inspect eigenvalues (values > 1) [17, 18] and estimate exploratory factor models (both one and two correlated factors models) and a bifactor measurement model. When there is potential multidimensionality, as is the case here, the bifactor measurement model is particularly useful because it partitions item variance into its unique sources [19]—i.e., it estimates the amount of variance that is common to all items versus the amount that is uniquely shared among smaller groups or subsets of items. The bifactor model disentangles these sources of variance in order to help determine whether the data are ‘essentially unidimensional’ [20, 21]—i.e., primarily reflecting one common construct—which can thus justify the use of a unidimensional item response theory (IRT) model [22, 23]. Further, the bifactor measurement model allows for the computation of statistical indices which provide additional information about the adequacy and appropriateness of using a total score [24]. Specifically, we compute omega hierarchical (omegaH), [25] a model-based reliability estimate of the proportion variance in total scores that is explained by the general factor. Omega hierarchical can also be computed on subscale scores (omegaHS) after controlling for the general factor to determine whether any unique reliable variance remains in subscale scores after controlling for a general factor. These reliability indices are evaluated according to conventional criteria for research (acceptable: 0.70 – 0.79; good: 0.80 – 0.89; excellent: ≥ 0.90) [26]. We also computed explained common variance (ECV) [27] which indexes the proportion of total variance (general plus specific) that is explained by the general factor alone. To evaluate the final results from the exploratory models, we estimated a confirmatory factor model and assessed it using traditional model fit indices such as the Root Mean Square Error of Approximation (RMSEA ≤ 0.08), [28] Comparative Fit Index (CFI ≥ 0.95), [29] and Standardized Root Mean Residual (SRMR ≤ 0.08) [29].

Assuming essential unidimensionality is met, we then fit a graded response model (GRM), [30] the most common IRT model for ordered item responses. The GRM, like other IRT models, specifies the relationship between a person’s responses to a set of items and the latent trait or construct being measured by the items, in this case, impact of chronic pain (hereafter referred to as impact). The purpose of this IRT model is to estimate item parameters to characterize the relationship between the items and the underlying construct being measured, in this case, impact. In the GRM there is one discrimination (i.e., slope) parameter and between category threshold (i.e., location) parameters for one less than the number of response categories (e.g., 5 categories = 4 location parameters). The discrimination or slope parameter reflects how well the item relates to the underlying construct (like a factor loading or item-total correlation) and thus how well an item is able to differentiate among individuals at different levels of the construct continuum. Generally speaking, higher slopes are desirable and indicate better discrimination [31]. Location parameters reflect spacing of the item responses across the construct continuum and the point on the construct where a respondent has a 50% chance of choosing a particular category or higher. Thus, the more chronic pain impact a person is experiencing, the more likely they are to endorse higher response categories. All analyses were conducted in R [32] using the psych [33] package for descriptive and exploratory factor analyses, lavaan [34] for confirmatory factor analysis, and mirt [35] for IRT.

To further evaluate the construct validity of the ISS, we examined how the ISS, and its components, were associated with the ODI total score and its individual components. We also evaluated discriminant validity by examining associations between the ISS and the PROMIS-29 domains.

## Results

Item descriptive statistics and correlations are presented in Tables 1 and 2, respectively. Cronbach’s alpha for the 9 items was excellent (α = 0.93) with item-total correlations ranging from 0.64 to 0.84. Item-total correlations were higher for PI items relative to the pain intensity and PF items (Table 1). All items were significantly (*p* < 0.0001) and positively correlated with one another with values ranging from 0.36 to 0.85 (Table 2). Not surprisingly, item correlations tended to be stronger between items within each scale (PF: *r*’s = 0.54 to 0.73; PI: *r*’s = 0.56 to 0.85). Further, pain intensity was more strongly associated with PI items (*r*’s = 0.56 to 0.67) than PF items (*r*’s = 0.36 to 0.43). The average item correlation was 0.58.

**Table 1:** Item means and item-rest correlations for ISS items

Items	M(SD)	Item-total correlation
Physical Function		
1. Are you able to do chores such as vacuuming or yard work? (1–5)	2.1 (0.97)	0.68
2. Are you able to go up and down stairs at a normal pace? (1–5)	1.9 (0.98)	0.65
3. Are you able to go for a walk of at least 15 min? (1–5)	1.6 (0.90)	0.65
4. Are you able to run errands and shop? (1–5)	1.6 (0.78)	0.73
Pain Interference		
5. How much did pain interfere with your day-to-day activities? (1–5)	2.3 (0.94)	0.79
6. How much did pain interfere with work around the home? (1–5)	2.3 (1.02)	0.84
7. How much did pain interfere with your ability to participate in social activities? (1–5)	1.8 (1.00)	0.76
8. How much did pain interfere with your household chores? (1–5)	2.1 (1.01)	0.82
Pain Intensity		
9. How would you rate your pain on average? (0–10)	3.9 (2.05)	0.64

Cronbach’s α = 0.93

**Table 2:** Product-moment correlations among items comprising the ISS

	Chores	Stairs	Walk15	Errands	Interfere daily	Interfere home	Interfere social	Interfere chores
1. Chores	-							
2. Stairs	0.59	-						
3. Walk15	0.54	0.65	-					
4. Errands	0.60	0.64	0.73	-				
5. Interfere daily	0.51	0.45	0.45	0.55	-			
6. Interfere home	0.58	0.49	0.48	0.56	0.85	-		
7. Interfere social	0.48	0.47	0.49	0.57	0.74	0.75	-	
8. Interfere chores	0.62	0.52	0.50	0.57	0.74	0.85	0.75	-
9. Pain intensity	0.43	0.39	0.36	0.43	0.67	0.66	0.56	0.62

Average inter-item correlation = 0.58

Eigenvalues and a scree plot indicate the presence of a strong primary dimension (eigenvalue = 5.67) and a possible second dimension (eigenvalue = 1.15), but all other eigenvalues were less than 1.0. Based on these results, item correlations described above, and consistent with the theoretical structure, we estimated exploratory factor analytic models (EFAs) for a one factor and two correlated factors model as well as a bifactor model. Results are presented in Table 3.

**Table 3:** Exploratory factor analysis factor loadings for one factor, two correlated factors, and bifactor model

	1 factor	2 factors		Bifactor		
Item #	𝜆	PII-𝜆	PF-𝜆	Gen—𝜆	PII—𝜆	PF -𝜆
1. Chores	0.70	-	0.52	0.64	-	0.30
2. Stairs	0.67	-	0.77	0.65	-	0.44
3. Walk15	0.67	-	0.89	0.67	-	0.50
4. Errands	0.75	-	0.80	0.72	-	0.45
5. Interfere daily	0.83	0.93	-	0.72	0.53	-
6. Interfere home	0.89	0.97	-	0.77	0.55	-
7. Interfere social	0.80	0.74	-	0.69	0.42	-
8. Interfere chores	0.87	0.82	-	0.75	0.46	-
9. Pain intensity	0.68	0.73	-	0.58	0.41	-

Factor loadings are denoted (𝜆)

*Gen* =General factor (i.e., impact), *PII* =Pain interference/intensity factor, *PF* =Physical function factor. EFA models estimated using minimum residual extraction with an oblimin rotation. Cross-loading values (< .20) are denoted (-) for ease of interpretation

**Fig. 1:**
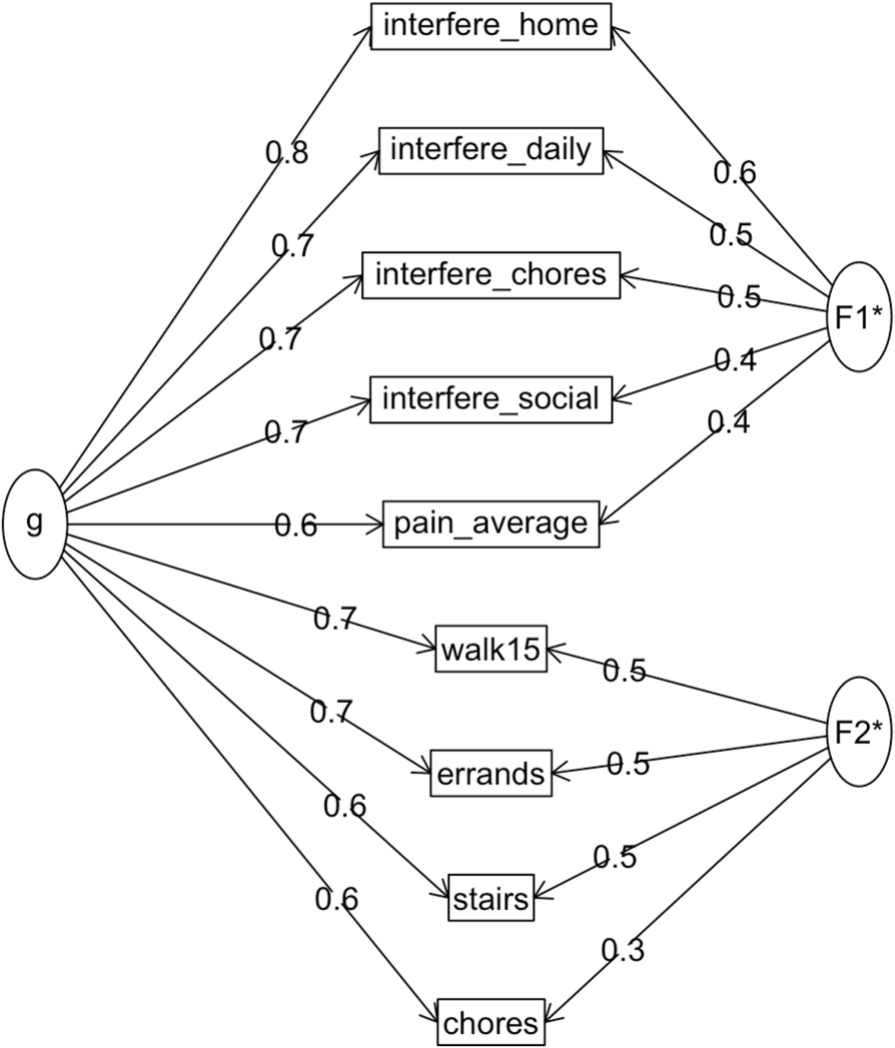
Bifactor measurement model demonstrating one general factor (g) underlying all items and two group factors consisting of four pain interference and one pain intensity item (F1) and four physical function items (F2)

In the one factor EFA, factor loadings (𝜆) ranged from 0.67 to 0.89. In the two correlated factors model, items partitioned into PF (𝜆= 0.52 to 0.89) and PI + pain intensity (𝜆 = 0.73 to 0.97) with a 0.68 correlation between factors. In the bifactor model (Fig. 1), all items loaded strongly on the general factor (𝜆 = 0.58 to 0.77) and corresponding group factors (PF: 𝜆 = 0.30 to 0.50; PI + intensity: 𝜆 = 0.41 to 0.55). OmegaH for general factor scores was 0.73, meeting the threshold for acceptable reliability. After partitioning out general factor variance, reliability was extremely poor for the subscale scores (omegaHS was 0.25 for PF subscale scores and 0.29 for PI + intensity subscale scores) implying very little meaningful variance being captured by the subscales. Lastly, ECV was 69%, indicating that over two-thirds of the common variance was explained by the general factor. A confirmatory factor model representing the bifactor structure fit the data well (RMSEA = 0.065 (CI: 0.056–0.075); SRMR = 0.018; CFI = 0.989) and was an improvement over the fit of the two correlated factors (RMSEA = 0.105 (CI: 0.097, 0.114), SRMR = 0.038, CFI = 0.958) and one factor (RMSEA = 0.203 (CI: 0.195, 0.211), SRMR = 0.087, CFI = 0.840) confirmatory factor models. Taken together, these results indicate that scores primarily reflect one underlying construct and that, once controlling for the general factor, very little unique reliable variance remained in subscale scores, providing support for the use of ISS total scores. These results also suggest that the 9-item scale is sufficiently unidimensional for IRT analyses.

Item parameters from the IRT model are presented in Table 4. Item slope parameters (measures of how well the item relates to the underlying construct) ranged from 1.4 to 7.2. The location parameters for all items spanned a wide range from -2.7 to 4.5 standard deviations (SD) on the impact continuum, indicating a good representation of varying levels of the construct. While all slopes were good, the largest slope (7.2) was markedly higher than the rest. This can often be an indication of possible local dependencies in the data (i.e., strong correlation between items after accounting for underlying trait) [36]. Further inspection revealed that there was a significant local dependence between this item (How much did pain interfere with work around the home?) and another item (How much did pain interfere with your household chores?)*.* Based on item content, it was apparent that these two items were redundant, explaining the correlation between these two items after accounting for the underlying trait (i.e., impact). As such, we removed the item with the largest slope and re-estimated the IRT model on the remaining eight items.

**Table 4:** Graded Response Model slope (a) and location (b) parameters for items comprising the 9-item ISS

	Slope	Location parameters									
Item	a	b1	b2	b3	b4	b5	b6	b7	b8	b9	b10
1. Chores	1.75	-0.79	0.58	1.97	3.02						
2. Stairs	1.43	-0.21	1.14	2.39	3.38						
3. Walk15	1.60	0.44	1.48	2.51	3.43						
4. Errands	1.98	0.26	1.49	2.75	4.07						
5. Interfere daily	4.28	-0.98	0.32	1.31	2.30						
6. Interfere home	7.20	-0.74	0.36	1.17	2.00						
7. Interfere social	3.22	-0.08	0.81	1.67	2.41						
8. Interfere chores	4.67	-0.56	0.54	1.39	2.07						
9. Pain intensity	1.86	-2.73	-1.57	-0.71	-0.07	0.44	0.96	1.61	2.44	3.55	4.54

**Fig. 2:**
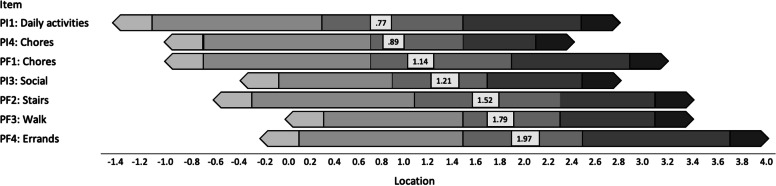
Item ranking from lowest to highest based on average location parameter. Physical function items are denoted PF and pain interference as PI. Vertical lines between shaded segments reflect threshold parameters (b1-b4). Superimposed boxes display the average location parameter for each item

Item parameters for the 8-item scale that corrects for this local dependence are presented in Table 5. Slope parameters were high for all items and ranged from 1.7 – 3.8 with no extreme values as seen previously. This indicates that all items were strong indicators of impact as well as able to highly differentiate between individuals at varying levels of the impact continuum. That said, there was variability in item slopes such that PI items were more discriminating (3.2 to 3.8) compared to pain intensity and PF items (1.7 to 2.4). Location parameters for all items were similar to the 9-item IRT model and spanned a wide range of the impact continuum (-2.8 to 4.6 SD). There was also variability in location parameters indicating some overall differences in item difficulty. For each item, the average location parameter was computed and is presented graphically in Fig. 2. Mean locations ranged from 0.77 to 1.97 SD. The two items with lowest average locations asked about pain interference in daily activities and chores, indicating that even people with low overall impact scores are likely to experience these two aspects of pain interference. In contrast, the three items with the highest average location parameters asked about physical function involving running errands, walking for 15 min, and walking up or down stairs, indicating that persons unable to do these activities are experiencing a higher overall impact.

**Table 5:** Graded Response Model slope (a) and location (b) parameters for items comprising the 8-item ISS

	Slope	Location parameters									
Item	a	b1	b2	b3	b4	b5	b6	b7	b8	b9	b10
1. Chores	1.94	-0.76	0.56	1.89	2.87						
2. Stairs	1.70	-0.20	1.05	2.18	3.06						
3. Walk15	1.93	0.40	1.35	2.29	3.10						
4. Errands	2.43	0.24	1.39	2.54	3.70						
5. Interfere daily	3.29	-1.04	0.34	1.38	2.40						
7. Interfere social	3.16	-0.08	0.83	1.69	2.42						
8. Interfere chores	3.83	-0.58	0.56	1.44	2.13						
9. Pain intensity	1.79	-2.79	-1.61	-0.73	-0.08	0.45	0.97	1.64	2.49	3.63	4.66

The item parameters from the 8-item IRT model were also used to generate several plots showing substantial spread of item responses and adequate representation at all levels of the impact continuum (see Supplemental Materials). Also, test information (or measurement precision) for the 8-item scale yielded a reliability greater than 0.70 from 2 SDs below the mean to 5 SDs above the mean of the impact continuum. Further, the marginal reliability was 0.90. Raw scores from the 8-item ISS were almost perfectly correlated (*r* = 0.997) with the original 9-item version indicating that virtually no information was lost by eliminating the redundant item.

As a construct validation check, we examined the association between ODI and ISS scores given that it would be anticipated, if the ISS was functioning as intended, that scores would be correlated. As expected, higher ISS scores were strongly and positively associated with higher ODI scores (*r* = 0.82, *p* < 0.001) thus providing support for the validity of the ISS. We also inspected how the ISS components (PI, PF, pain intensity) were associated with the ODI score and its individual elements. The ODI was strongly correlated with PF (*r* = 0.72), PI (*r* = 0.71), and moderately correlated with pain intensity (*r* = 0.59). Correlations between PF and ODI elements ranged from 0.33 (sitting) to 0.69 (walking). For PI, the correlations with ODI elements ranged from 0.36 (sitting) to 0.65 (social life). Pain intensity correlations with ODI elements ranged from 0.33 (walking) to 0.59 (pain intensity). Interestingly, but not surprisingly, the strongest correlations (≥ 0.50) between PI and ODI elements were for items on pain intensity, personal care, traveling, sex life, and social life. For PF, the strongest correlations were with items on personal care, social life, lifting, standing, and walking. Thus, some of the strongest correlations with PI pertained to interference in the ability to participate in activities whereas for PF the strongest correlations were capturing elements of physical limitations.

As a final evaluation of the validity and utility of the ISS, we compared associations between the ISS and PROMIS-29 domains as well as with the ODI score to further understand the specificities of the ISS. Correlations between the ISS and PROMIS-29 domains were lower for anxiety (*r* = 0.38), depression (*r* = 0.41), and sleep (*r* = 0.40); moderate for fatigue (*r* = 0.51); and highest for social roles (*r* = -0.74), pain intensity (*r* = 0.77), physical function (*r* = 0.79), and pain interference (*r* = 0.88). Further, as would be expected, correlations were low to moderate between the ODI score and remaining PROMIS domains (anxiety: *r* = 0.32, depression: *r* = 0.35, fatigue: *r* = 0.41, sleep: *r* = 0.37, social: *r* = -0.66) compared to the strong correlation with the ISS (*r* = 0.82).

## Discussion

This study investigated the dimensional structure of the ISS items to assess the appropriateness of scoring these items as one total score composite reflecting the impact of chronic low back pain. Preliminary examination via exploratory factor analysis provided initial support for the presence of a strong underlying dimension and possibly a lesser, additional dimension. A bifactor measurement model found one general underlying dimension as well as two smaller group factors consistent with the PROMIS domains—one containing the physical function items and one containing the pain interference and intensity items. The advantage of the bifactor model is the ability to partition item variance into its specific sources, namely, general and group factors and to test the reliability of each. We found that general impact factor scores were reliable according to conventional criteria and that there was little if any reliable variance remaining in group factors scores after the general factor reliability was accounted for. The ISS was, therefore, found to be essentially unidimensional, thus providing support for IRT analysis as well as the use of its total score and its use in practice as a general measure of the impact of CLBP.

The IRT model for the 9-item ISS indicated that items were generally good indicators of impact and that there was noticeable representation across the impact continuum. However, there was concern over the large magnitude of one of the item slopes that was outside the range of the other items and likely due to highly redundant item content. As such, the exclusion of the ‘problematic’ item and re-estimation of the IRT model provided support for a psychometrically sound reduced eight item version. For the 8-item ISS, not only were the individual items excellent, the degree of information, precision, and reliability were also noteworthy.

Taken together, this study not only supports the use of the 9-item ISS but also offers the option of a shorter 8-item alternative which corrects for local dependence. As noted above, while use of the 9-item version is supported, it contains two items with relatively redundant content. As such, the ISS total score is doubly counting pain interference on work around the house (i.e., chores). The shorter 8-item version, however, eliminates the redundancy such that pain interference with household chores contributes to the ISS total score only once. If the goal is to form an impact score that covers relatively distinct aspects of chronic pain impact, the 8-item option may be more suitable. Further, use of the 8-item version is also supported given that scores were nearly perfectly correlated with those computed from the 9-item version.

The 2015 NPS report defined high-impact chronic pain as being: “associated with substantial restriction of participation in work, social, and self-care activities for six months or more” [3], p11. While not prescriptive, the IRT results provide more nuance and detail to this definition and help clinicians better understand and unpack the impact of chronic pain on their patients. Aspects of pain interference appeared more prevalent at lower levels of the impact continuum. For instance, the lowest average location parameters were for pain interference with daily activities and chores indicating that, in general, these items are likely to manifest at lower levels of impact. On the other hand, elements of physical functioning appeared to be affected at higher levels of the impact continuum. Ability to run errands, walk for 15 min, and climb stairs had much higher average location parameters. On average, impact needed to be more severe for pain to affect physical function in these areas. These findings may suggest that certain activity limitations (i.e., interference) might emerge before participation restrictions (i.e., function).

Validity analyses provided support for the utility of the ISS such that, as expected, the ISS, and its components, were strongly associated with the ODI total score. Overall the association between the ISS and ODI total score was strongest; however, at the component level, PF and PI were more strongly correlated with the ODI total score than pain intensity. PF was most strongly associated with ODI elements measuring physical limitations, and PI was most strongly associated with ODI elements capturing an inability to participate in activities. Additionally, correlations between the ISS and other PROMIS-29 domains indicated that, while related, the ISS was distinct.

This study had the benefit of a large dataset containing the PROMIS-29 v2.1 items required to calculate the ISS on patients with CLBP, but also has limitations. The approach of using a sample of patients with CLBP was purposeful as the ISS measure was proposed for use in patients with CLBP. However, these results may not generalize to other pain populations (e.g., patients with headache or hip pain). The sample was also of patients using chiropractic for their CLBP and was predominantly made up of patients identifying as White and female, which may further limit the generalizability of these findings. Therefore, the results in this study should be replicated in other samples. Our analyses should also be evaluated using longitudinal data to determine whether the differences seen in respondents across the impact continuum represent the differences that would be seen in individual patients as their ISS scores improve or worsen.

## Conclusions

The ISS was proposed as a measure of the impact of chronic low back pain and is made up of the combination of two four-item PROMIS scales and one single item. This study adds to other analyses of the ISS by examining its dimensionality and the appropriateness of its scoring. We found that it was sufficiently unidimensional and that use of a total score was appropriate. IRT analysis showed that items were good indicators of impact and provided information across a wide range of the impact continuum. Moreover, IRT modeling provided support for a shorter 8-item version of the ISS which eliminates item content redundancy.

The IRT results also suggest that as the impact of chronic pain increases different aspects of pain interference occur before losses in physical function. Future studies should examine how individuals’ ISS scores change over time and whether change is consistent with this these findings.

## Supplementary Information

**Additional file 1: Figure 3. **Category response curves for the 8 items comprising the ISS. The impact continuum is denoted ($\mathrm{\theta}$) and has a mean of 0 and standard deviation of 1. Curves demonstrate the probability of choosing a specific category for a given score on the impact continuum. **Figure 4.** Item information for the 8 items comprising the ISS. The impact continuum is denoted ($\mathrm{\theta}$) and has a mean of 0 and standard deviation of 1. Curves indicate where on the impact continuum information is greatest and precision at a maximum. **Figure 5. **Test Information/ Standard Errors (a) and Reliability (b). The impact continuum is denoted ($\mathrm{\theta}$) and has a mean of 0 and standard deviation of 1. Peaks of curves indicate where on the impact continuum (a) information, precision, and (b) reliability are greatest. In figure b the horizontal line at 0.70 corresponds to the conventional threshold for acceptable reliability.

## Data Availability

The datasets generated and/or analyzed during the current study are not publicly available due to lack of participant consent to share their data but are available from the corresponding author on reasonable request.

## Abbreviations

CERC: The Center for Excellence in Research for Complementary and Integrative Health
CFI: Comparative Fit Index
CLBP: Chronic Low Back Pain
CNP: Chronic Neck Pain
ECV: Explained Common Variance
EFA: Exploratory Factor Analysis
GRM: Graded Response Model
IOM: Institute of Medicine
IRT: Item Response Theory
ISS: Impact Stratification Score
LBP: Low Back Pain
NPS: National Pain Strategy
ODI: Oswestry Disability Index
PF: Physical Function
PI: Pain Interference
PROMIS: Patient-Reported Outcomes Measurement Information System
PROMIS-29: 29-Item PROMIS Profile Measure
RMDQ: Roland-Morris Disability Questionnaire
RMSEA: Root Mean Square Error of Approximation
RTF: Research Task Force
SRMR: Standardized Root Mean Residual
