# Supplementary material for: Unpacking the impact of chronic pain as measured by the impact stratification score
Source: BMC Musculoskelet Disord. 2022 Sep 23;23:884. doi: 10.1186/s12891-022-05834-4 (PMC9503239; doi:10.1186/s12891-022-05834-4)
Supplement: Supplementary file 1 — Additional file 1: Figure 3. Category response curves for the 8 items comprising the ISS. The impact continuum is denoted ($\mathrm{\theta}$θ) and has a mean of 0 and standard deviation of 1. Curves demonstrate the probability of choosing a specific category for a given score on the impact continuum. Figure 4. Item information for the 8 items comprising the ISS. The impact continuum is denoted ($\mathrm{\theta}$θ) and has a mean of 0 and standard deviation of 1. Curves indicate where on the impact continuum information is greatest and precision at a maximum. Figure 5. Test Information/ Standard Errors (a) and Reliability (b). The impact continuum is denoted ($\mathrm{\theta}$θ) and has a mean of 0 and standard deviation of 1. Peaks of curves indicate where on the impact continuum (a) information, precision, and (b) reliability are greatest. In figure b the horizontal line at 0.70 corresponds to the conventional threshold for acceptable reliability. [file 12891_2022_5834_MOESM1_ESM.docx]

**Supplemental Figures**


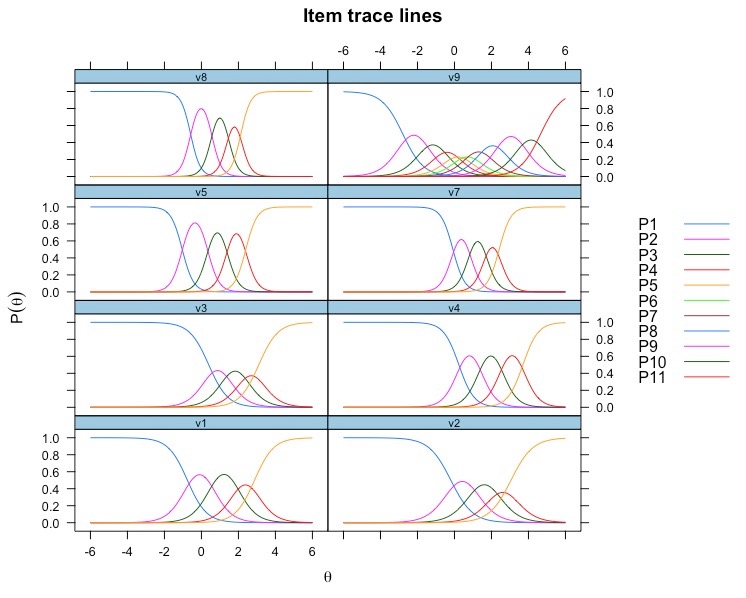


Figure 3. Category response curves for the 8 items comprising the ISS. The impact continuum is denoted (θ) and has a mean of 0 and standard deviation of 1. Curves demonstrate the probability of choosing a specific category for a given score on the impact continuum.


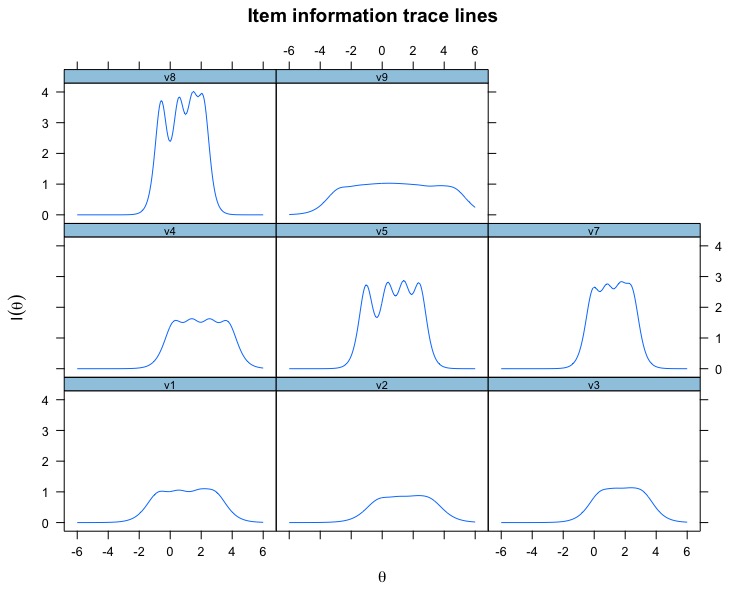


Figure 4. Item information for the 8 items comprising the ISS. The impact continuum is denoted (θ) and has a mean of 0 and standard deviation of 1. Curves indicate where on the impact continuum information is greatest and precision at a maximum.


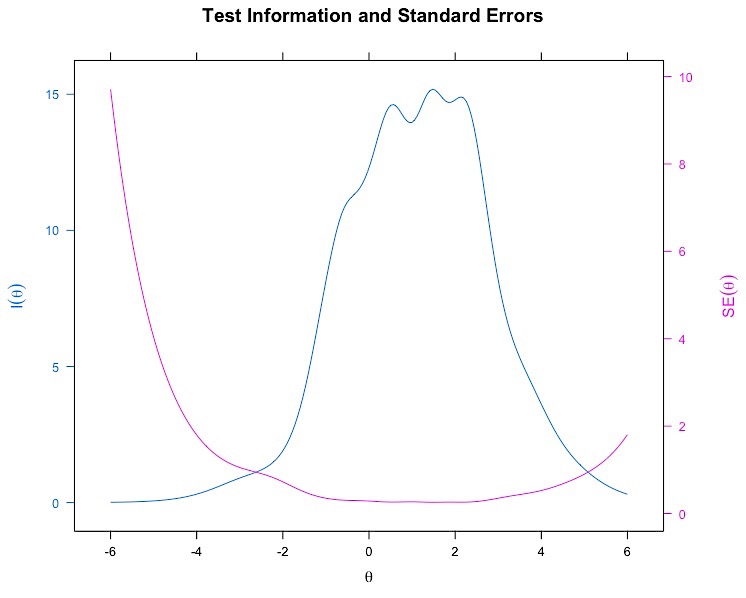


(a)


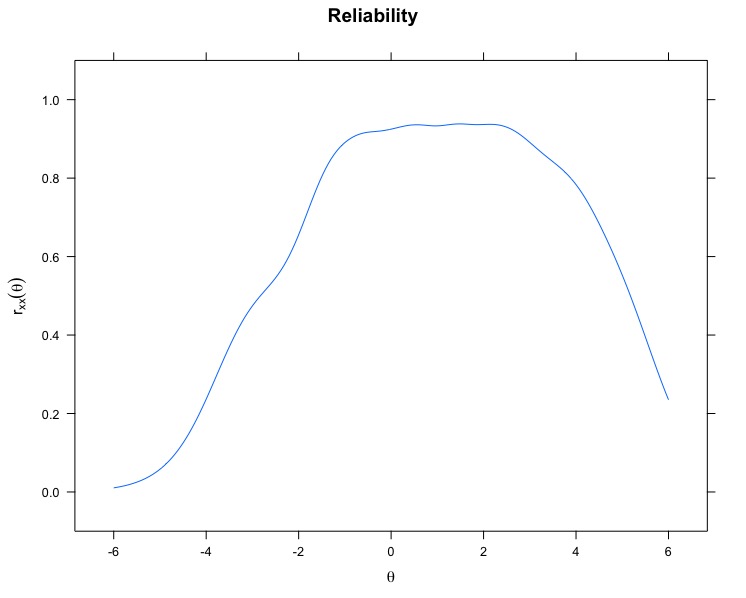


(b)

Figure 5. Test Information/ Standard Errors (a) and Reliability (b). The impact continuum is denoted (θ) and has a mean of 0 and standard deviation of 1. Peaks of curves indicate where on the impact continuum (a) information, precision, and (b) reliability are greatest. In figure b the horizontal line at 0.70 corresponds to the conventional threshold for acceptable reliability.
